# Supplementary material for: Habitat Elevation Shapes Microbial Community Composition and Alter the Metabolic Functions in Wild Sable (Martes zibellina) Guts
Source: Animals (Basel). 2021 Mar 18;11(3):865. doi: 10.3390/ani11030865 (PMC8002971; doi:10.3390/ani11030865)
Supplement: Supplementary file 1 [file animals-11-00865-s001.pdf]

SUPPLEMENTARY MATERIALS

Habitat Elevation Shapes Microbial Community Composition and Enhances the Metagenomic Functions in Wild Sable (*Martes Zibellina*) Guts

Lantian Su, Xinxin Liu, Guangyao Jin, Yue Ma, Haoxin Tan, Muhammed Khalid, Martin Romantschuk, Shan Yin, Nan Hui

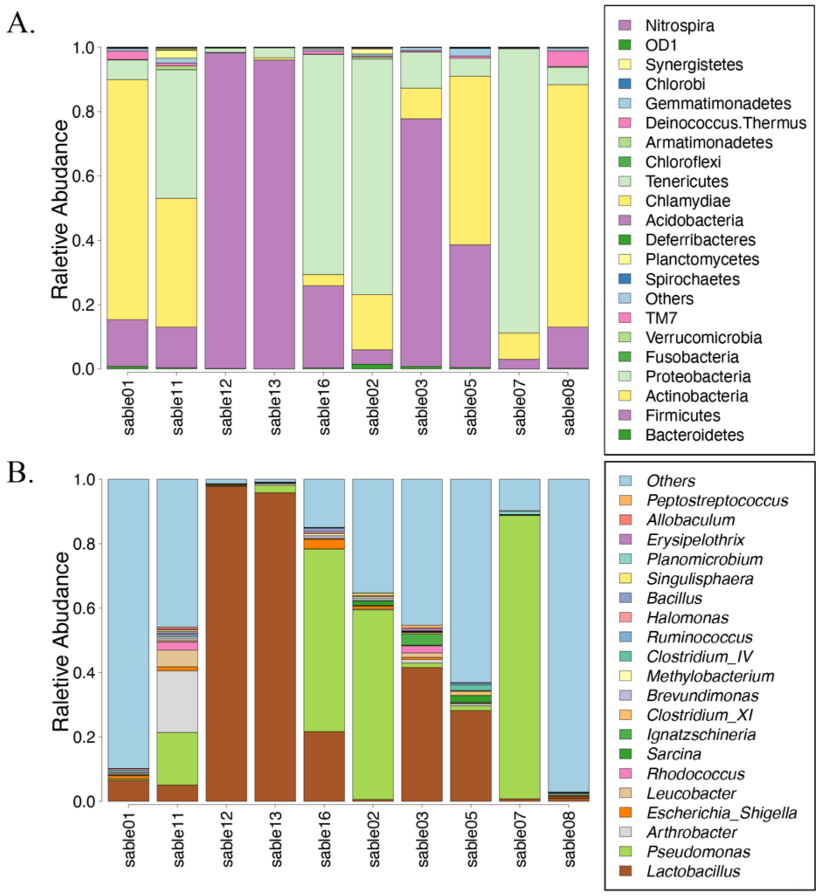

FIGURE S1. Gut microbial composition of wild sables. **(A)** Relative abundance of gut microbiota at the phylum level of each sable gut microbiome; **(B)** Top 20 bacterial classified genera in wild sable gut. The remaining genera are summed and classified as *Others*.

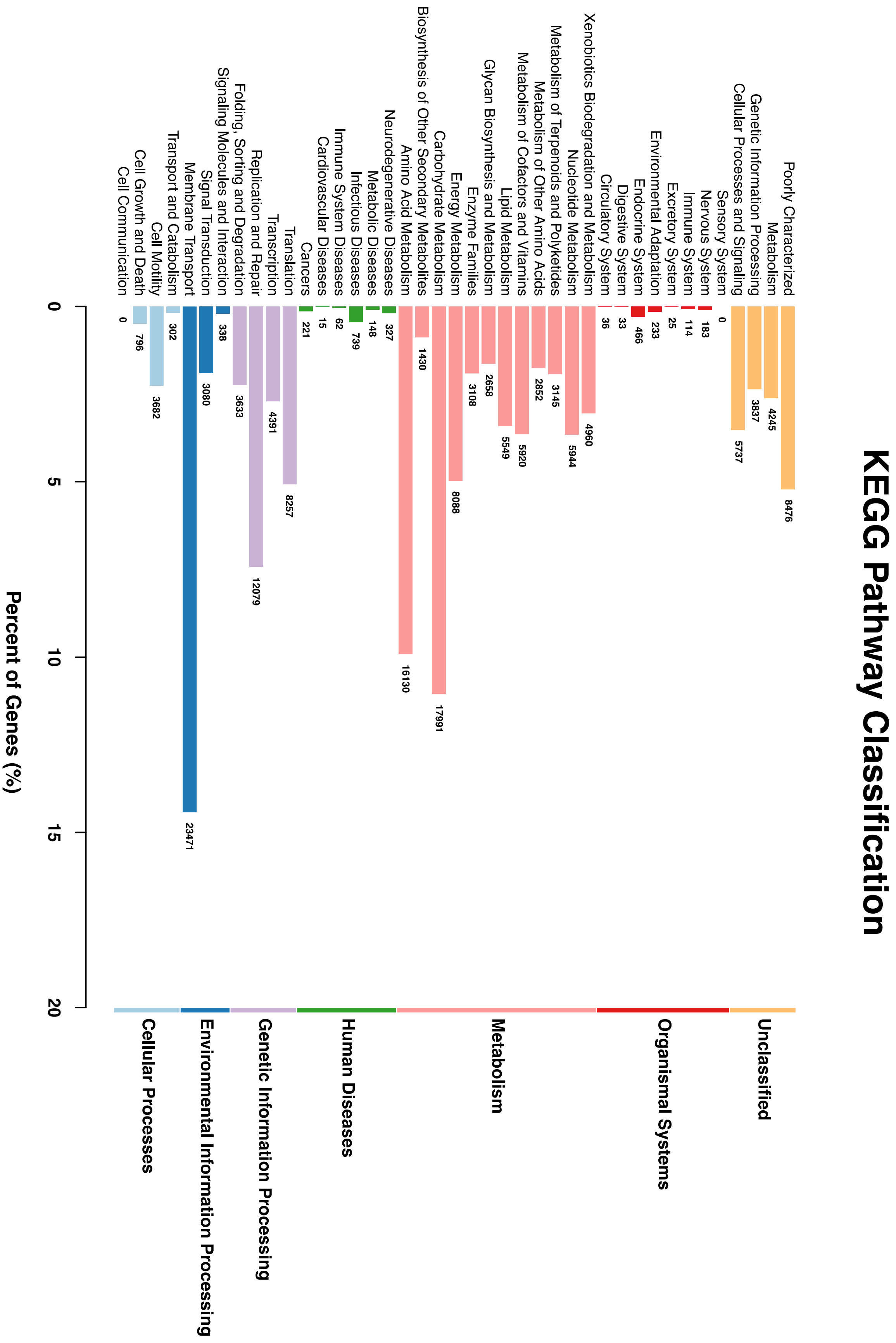

FIGURE S2. Functional gene benchmark the wild sable gut metagenome at KEGG level 2. Functional genes that belong to different groups at KEGG level 1 are distinguished by different color.

TABLE S1. DNA-based qPCR primer of each gene from gut samples

Gene	Description	Forward primer (5' - 3')	Reverse primer (5' - 3')
lpxB	Lipid A disaccharide synthase	CCGAAGTGCGTTTTGTCCTG	AGAATCCAGAACGTCAGGCG
mlaE	Lipid asymmetry maintenance ABC transporter permease subunit MlaE	TAATGGACCGAATCGGCCTG	CGGAGTGCAGCTGCTTGATC
uidA	Beta-glucuronidase	TTCAACCGTCTGGAAGTCCG	GCTTGCGCGATGAAATGGAT

TABLE S2. DNA-based qPCR programs and reagents

Reagent	Volume (µL)	qPCR program
H_2O	6	1. 95°C 15 min
Master Mix	10	2. 94°C 10 sec
Primer forward	1	3. 57°C 20 sec
Primer reverse	1	4. 72°C 30 sec
Template	2	5. Plate read
Total	20	6. Go to line 2 for 34 more cycles
-	-	7. Incubate at 95°C for 10 sec
-	-	8. Melting curve from 65°C to 95°C, increment 0.5°C 5 sec

TABLE S3. Correlation analyses between phyla relative abundance and environmental factors

Genera	Envrionmental factors	*p*	r
Lactobacillus	Average altitude	0.031	-0.68
Pseudomonas	Average altitude	0.035	0.67
Planomicrobium	Average altitude	0.037	0.66
Kurthia	Average altitude	0.033	-0.67
Olsenella	Average altitude	0.030	0.68
Turicibacter	Canopy density	0.047	-0.64
Rhodobacter	Canopy density	0.047	0.64
Humicoccus	Canopy density	0.048	0.64
Flavobacterium	Canopy density	0.042	0.65
Haemophilus	Canopy density	0.019	0.72
Abiotrophia	Canopy density	0.023	-0.70
Pasteuria	Canopy density	0.044	0.64
Sediminibacterium	Canopy density	0.004	0.82
Bdellovibrio	Canopy density	0.044	0.64
Rhodomicrobium	Canopy density	0.012	0.75
Shewanella	Canopy density	0.032	0.68
Parachlamydia	Canopy density	0.039	0.66
Atopobium	Canopy density	0.019	0.72
Coxiella	Canopy density	0.027	0.69
Allobaculum	Coverage	0.023	0.70

Genera	Envrionmental factors	*p*	r
Arthrobacter	Coverage	0.024	0.70
Leucobacter	Coverage	0.030	0.68
Sutterella	Coverage	0.022	0.71
Kaistia	Coverage	0.018	0.72
Mycobacterium	Coverage	0.038	0.66
Janthinobacterium	Coverage	0.027	0.69
Lactococcus	Coverage	0.022	0.71
Turicibacter	Coverage	0.043	-0.65
Labrys	Coverage	0.037	0.66
Corynebacterium	Coverage	0.022	0.71
Hyphomicrobium	Coverage	0.031	0.68
Staphylococcus	Coverage	0.025	0.70
Rhodobacter	Coverage	0.029	0.69
Humicoccus	Coverage	0.020	0.72
Schlesneria	Coverage	0.032	0.68
Flavobacterium	Coverage	0.021	0.71
Aeromicrobium	Coverage	0.022	0.71
Nocardioides	Coverage	0.038	0.66
Haemophilus	Coverage	0.009	0.77
Paenibacillus	Coverage	0.024	0.70
Morganella	Coverage	0.026	0.69
Gemmata	Coverage	0.021	0.71
Abiotrophia	Coverage	0.015	-0.74
Williamsia	Coverage	0.020	0.71
Gp16	Coverage	0.013	0.75
Aquicella	Coverage	0.043	0.65
Neochlamydia	Coverage	0.046	0.64
Pasteuria	Coverage	0.016	0.73
Sediminibacterium	Coverage	0.030	0.68
Gemmatimonas	Coverage	0.040	0.65
Bdellovibrio	Coverage	0.016	0.73
Marmoricola	Coverage	0.021	0.71
Zavarzinella	Coverage	0.020	0.71
Pseudonocardia	Coverage	0.021	0.71
Actinoplanes	Coverage	0.047	0.64
Verrucomicrobium	Coverage	0.022	0.71
Rhodomicrobium	Coverage	0.009	0.77
Roseomonas	Coverage	0.031	0.68
Actinomadura	Coverage	0.022	0.71
Parachlamydia	Coverage	0.017	0.73
Amaricoccus	Coverage	0.022	0.71
Cohnella	Coverage	0.022	0.71

Genera	Envrionmental factors	*p*	r
Simplicispira	Coverage	0.044	0.65
Atopobium	Coverage	0.009	0.77
Myroides	Coverage	0.022	0.71
Tumebacillus	Coverage	0.022	0.71
Dietzia	Coverage	0.022	0.71
Smaragdicoccus	Coverage	0.022	0.71
Streptosporangium	Coverage	0.022	0.71
Camelimonas	Coverage	0.022	0.71
Coxiella	Coverage	0.010	0.76
Anaeromyxobacter	Coverage	0.022	0.71
Akkermansia	Fallen wood	0.010	0.77
Streptococcus	Fallen wood	0.038	0.66
Treponema	Fallen wood	0.006	0.80
Mucispirillum	Fallen wood	0.042	0.65
Brachyspira	Fallen wood	0.002	0.84
Propionibacterium	Fallen wood	0.049	0.63
Granulicatella	Fallen wood	0.004	0.82
Adhaeribacter	Fallen wood	0.002	0.84
Gallicola	Fallen wood	0.002	0.85
Photobacterium	Fallen wood	0.002	0.85
Diaphorobacter	Fallen wood	0.030	0.68
Methylobacillus	Fallen wood	0.002	0.84
Achromobacter	Hiding cover	0.010	0.77
Geodermatophilus	Hiding cover	0.006	-0.80
Methylocystis	Hiding cover	0.007	-0.79
Delftia	Snow depth	0.033	-0.67

TABLE S4. Correlation analyses between functional genes and environmental factors

Functional genes	Environmental factors	*p*	r
General function prediction only	Average altitude	0.003	0.83
Glutamatergic synapse	Average altitude	0.004	0.82
Transcription factors	Average altitude	0.005	0.80
Other transporters	Average altitude	0.006	0.80
Glycan biosynthesis and metabolism	Average altitude	0.007	0.78
Bacterial secretion system	Average altitude	0.011	0.76
Arginine and proline metabolism	Average altitude	0.011	0.76
Cyanoamino acid metabolism	Average altitude	0.014	0.74
Biosynthesis and biodegradation of secondary metabolites	Average altitude	0.014	0.74
Vibrio cholerae pathogenic cycle	Average altitude	0.016	0.73
Sulfur relay system	Average altitude	0.019	0.72
Phosphonate and phosphinate metabolism	Average altitude	0.021	0.71
Energy metabolism	Average altitude	0.022	0.71

**Functional genes**	**Environmental factors**	***p***	**r**
Glyoxylate and dicarboxylate metabolism	Average altitude	0.026	0.69
Folate biosynthesis	Average altitude	0.027	0.69
Pathways in cancer	Average altitude	0.028	0.69
Protein folding and associated processing	Average altitude	0.029	0.68
Biosynthesis of siderophore group nonribosomal peptides	Average altitude	0.031	0.68
Ribosome biogenesis in eukaryotes	Average altitude	0.032	0.68
Epithelial cell signaling in Helicobacter pylori infection	Average altitude	0.035	0.67
Biosynthesis of type II polyketide products	Average altitude	0.035	0.67
Melanogenesis	Average altitude	0.035	0.67
Cell motility and secretion	Average altitude	0.037	0.66
Porphyrin and chlorophyll metabolism	Average altitude	0.037	0.66
Pertussis	Average altitude	0.038	0.66
Systemic lupus erythematosus	Average altitude	0.038	0.66
Lipid metabolism	Average altitude	0.040	0.66
Nitrogen metabolism	Average altitude	0.040	0.65
Staphylococcus aureus infection	Average altitude	0.049	-0.63
Fructose and mannose metabolism	Average altitude	0.048	-0.64
Chromosome	Average altitude	0.047	-0.64
Purine metabolism	Average altitude	0.045	-0.64
Phosphatidylinositol signaling system	Average altitude	0.045	-0.64
DNA replication proteins	Average altitude	0.045	-0.64
Glycerolipid metabolism	Average altitude	0.044	-0.64
Glycosyltransferases	Average altitude	0.041	-0.65
DNA repair and recombination proteins	Average altitude	0.041	-0.65
Carbohydrate digestion and absorption	Average altitude	0.039	-0.66
Bisphenol degradation	Average altitude	0.037	-0.66
Thiamine metabolism	Average altitude	0.032	-0.67
Ethylbenzene degradation	Average altitude	0.030	-0.68
Cytoskeleton proteins	Average altitude	0.029	-0.69
Mismatch repair	Average altitude	0.028	-0.69
Taurine and hypotaurine metabolism	Average altitude	0.026	-0.69
Linoleic acid metabolism	Average altitude	0.025	-0.70
Glycosphingolipid biosynthesis globo series	Average altitude	0.025	-0.70
D Alanine metabolism	Average altitude	0.022	-0.71
Restriction enzyme	Average altitude	0.021	-0.71
Glycolysis Gluconeogenesis	Average altitude	0.020	-0.72
Xylene degradation	Average altitude	0.019	-0.72
Drug metabolism other enzymes	Average altitude	0.019	-0.72
Streptomycin biosynthesis	Average altitude	0.018	-0.72
D Arginine and D ornithine metabolism	Average altitude	0.018	-0.72
Methane metabolism	Average altitude	0.014	-0.74
Other glycan degradation	Average altitude	0.013	-0.75

Functional genes	Environmental factors	*p*	r
RIG I like receptor signaling pathway	Average altitude	0.013	-0.75
Dioxin degradation	Average altitude	0.011	-0.76
Sphingolipid metabolism	Average altitude	0.011	-0.76
Base excision repair	Average altitude	0.010	-0.76
Amino acid metabolism	Average altitude	0.009	-0.77
Cell cycle Caulobacter	Average altitude	0.008	-0.78
Polycyclic aromatic hydrocarbon degradation	Average altitude	0.007	-0.78
Prenyltransferases	Average altitude	0.007	-0.79
Pyruvate metabolism	Average altitude	0.006	-0.79
Stilbenoid diarylheptanoid and gingerol biosynthesis	Average altitude	0.006	-0.80
Replication recombination and repair proteins	Average altitude	0.003	-0.82
Nucleotide metabolism	Average altitude	0.002	-0.85
Fatty acid elongation in mitochondria	Canopy density	0.005	0.81
Fatty acid elongation in mitochondria	Fallen wood	0.042	0.65
Cell cycle Caulobacter	Snow depth	0.047	-0.64
Glycosphingolipid biosynthesis ganglio series	Snow depth	0.026	-0.69
Fatty acid elongation in mitochondria	Vegetation Coverage	0.013	0.75
Glycosylphosphatidylinositol GPI anchor biosynthesis	Vegetation Coverage	0.022	0.71
Lipoic acid metabolism	Vegetation Coverage	0.024	0.70
Caffeine metabolism	Vegetation Coverage	0.026	0.69
Photosynthesis antenna proteins	Vegetation Coverage	0.027	0.69
Glycosphingolipid biosynthesis lacto and neolacto series	Vegetation Coverage	0.029	0.68
Clavulanic acid biosynthesis	Vegetation Coverage	0.031	0.68
Amino acid metabolism	Vegetation Coverage	0.032	0.67
Biosynthesis of 12 14 and 16 membered macrolides	Vegetation Coverage	0.042	0.65
Indole alkaloid biosynthesis	Vegetation Coverage	0.042	0.65
Calcium signaling pathway	Vegetation Coverage	0.045	0.64
Transcription factors	Vegetation Coverage	0.048	-0.64
Bacterial toxins	Vegetation Coverage	0.034	-0.67
Chaperones and folding catalysts	Vegetation Coverage	0.030	-0.68
Ribosome biogenesis in eukaryotes	Vegetation Coverage	0.027	-0.69
